# Advanced Optoelectronic Modeling and Optimization of HTL-Free FASnI_3_/C60 Perovskite Solar Cell Architecture for Superior Performance

**DOI:** 10.3390/nano14121062

**Published:** 2024-06-20

**Authors:** Tariq AlZoubi, Wasan J. Kadhem, Mahmoud Al Gharram, Ghaseb Makhadmeh, Mohamed A. O. Abdelfattah, Abdulsalam Abuelsamen, Ahmad M. AL-Diabat, Osama Abu Noqta, Bojan Lazarevic, Samer H. Zyoud, Bachar Mourched

**Affiliations:** 1 College of Engineering and Technology, American University of the Middle East, Egaila 54200, Kuwait; 2 Department of Scientific Basic Sciences, Faculty of Engineering Technology, Al-Balqa Applied University, Amman 11134, Jordan; 3 Department of Physics, School of Electrical Engineering and Information Technology (SEEIT), German Jordanian University, Amman 11180, Jordan; 4 General Education Department, Skyline University College, Sharjah P.O. Box 1797, United Arab Emirates; 5 Medical Imaging and Radiography Department, Aqaba University of Technology, Aqaba 910122, Jordan; 6 Department of Physics, Al-Zaytoonah University of Jordan, Amman 11733, Jordan; 7 MEU Research Unit, Middle East University, Amman 11831, Jordan; 8 Nonlinear Dynamics Research Center (NDRC), Department of Mathematics and Sciences, Ajman University, Ajman P.O. Box 346, United Arab Emirates

**Keywords:** numerical modeling, perovskite optimization, FASnI_3_, C60, solar cell, SCAPS, PSC optical properties

## Abstract

In this study, a novel perovskite solar cell (PSC) architecture is presented that utilizes an HTL-free configuration with formamide tin iodide (FASnI_3_) as the active layer and fullerene (C60) as the electron transport layer (ETL), which represents a pioneering approach within the field. The elimination of hole transport layers (HTLs) reduces complexity and cost in PSC heterojunction structures, resulting in a simplified and more cost-effective PSC structure. In this context, an HTL-free tin HC(NH_2_)_2_SnI_3_-based PSC was simulated using the solar cell capacitance simulator (SCAPS) within a one-dimensional framework. Through this approach, the device performance of this novel HTL-free FASnI_3_-based PSC structure was engineered and evaluated. Key performance parameters, including the open-circuit voltage (V_oc_), short-circuit current density (J_sc_), fill factor (FF), power conversion efficiency (PCE), I-V characteristics, and quantum efficiency (QE), were systematically assessed through the modulation of physical parameters across various layers of the device. A preliminary analysis indicated that the HTL-free configuration exhibited improved I-V characteristics, with a PCE increase of 1.93% over the HTL configuration due to improved electron and hole extraction characteristics, reduced current leakage at the back contact, and reduced trap-induced interfacial recombination. An additional boost to the device’s key performance parameters has been achieved through the further optimization of several physical parameters, such as active layer thickness, bulk and interface defects, ETL thickness, carrier concentration, and back-contact materials. For instance, increasing the thickness of the active layer PSC up to 1500 nm revealed enhanced PV performance parameters; however, further increases in thickness have resulted in performance saturation due to an increased rate of hole–electron recombination. Moreover, a comprehensive correlation study has been conducted to determine the optimum thickness and donor doping level for the C60-ETL layer in the range of 10–200 nm and 10^12^–10^19^ cm^−3^, respectively. Optimum device performance was observed at an ETL-C60 ultra-thin thickness of 10 nm and a carrier concentration of 10^19^ cm^−3^. To maintain improved PCEs, bulk and interface defects must be less than 10^16^ cm^−3^ and 10^15^ cm^−3^, respectively. Additional device performance improvement was achieved with a back-contact work function of 5 eV. The optimized HTL-free FASnI_3_ structure demonstrated exceptional photovoltaic performance with a PCE of 19.63%, V_oc_ of 0.87 V, J_sc_ of 27.86 mA/cm^2^, and FF of 81%. These findings highlight the potential for highly efficient photovoltaic (PV) technology solutions based on lead-free perovskite solar cell (PSC) structures that contribute to environmental remediation and cost-effectiveness.

## 1. Introduction

Organic–inorganic lead halide perovskite solar cells have attracted tremendous interest as next-generation solar cells, reaching a power conversion efficiency (PCE) of 26.1% [1,2,3,4]. Despite extensive research efforts to enhance the performance of perovskite solar cells (PSCs) with lead-based CH_3_NH_3_PbI_3_ as the absorbing material, the toxicity of lead has hindered the large-scale implementation of these solar devices [5,6]. Hence, there is a pressing need to explore alternative perovskite materials to overcome this limitation. Recently, attention has turned towards Pb-free alternatives, including tin-based PSCs, such as FASnI_3_, which exhibits intrinsically favorable optical band gaps, electronic band structures, high absorption coefficients, earth abundance, and a non-toxic nature, making it a promising candidate for next-generation PSCs [7,8,9,10,11]. Building on the stereochemical principles, replacing Pb^2+^ with Sn^2+^ in perovskite structures offers a promising pathway to enhance PSC stability. This activity results in deviations from the ideal octahedral configuration of metal–halide structures. Specifically, the stereochemical activity of Pb^2+^ distorts the PbI_6_ octahedron, favoring a tetragonal phase but introducing a high energy barrier for phase transitions and increasing susceptibility to degradation [8,12]. In contrast, replacing Pb^2+^ with Sn^2+^ in the perovskite structure is hypothesized to enhance stability [13,14]. This is due to the similarities in structural formation properties between Sn^2+^ and Pb^2+^, and the lower oxidation state of Sn^2+^ results in reduced stereochemical activity, potentially leading to a more stable perovskite crystalline structure and framework [15,16,17,18].

Over the past decade, the drive for high power conversion efficiency (PCE) has triggered extensive research activities into replacing lead with tin in perovskite solar cells. As such, numerous researchers have explored the use of CH_3_NH_3_SnI_3_ (MASnI_3_) as the active layer in perovskite solar cells. However, for achieving high efficiency in tin-based perovskite solar cells, formamidinium tin iodide FASnI_3_ has been predominantly utilized. The larger ionic radius of FA (CH(NH_2_)_2_) in FASnI_3_ leads to a reduced antibonding interaction between Sn-5s and I-5p orbitals compared to MASnI3, despite FASnI_3_’s adjustable conductivity. Research by the Milot group indicates that FASnI_3_ has superior carrier mobility, lower Auger recombination, and higher radiative bimolecular recombination rates compared to MASnI_3_. In 2015, Krishnamoorthy et al. were the first to report on FASnI_3_-based perovskite solar cells, achieving a power conversion efficiency (PCE) of 2.1% [19]. A wide range of techniques used to enhance the efficiency of lead-based perovskite solar cells have also been applied to their tin-based counterparts. To address the oxidation issue of SnX_2_, where Sn^2+^ ions convert to Sn^4+^ in air, Bian et al. [20] incorporated Sn powder into SnI_2_, which reduced Sn^4+^ ions back to Sn^2+^. This approach resulted in a maximum PCE of 6.75% in a p-i-n architecture for FASnI_3_-based solar cells. In 2019, Wu et al. [21] demonstrated a 10.1% power conversion efficiency by incorporating multi-conjugated Lewis’s base molecules to manage grain boundaries during the crystallization of FASnI_3_ films. This solar cell was constructed using ITO/PEDOT:PSS/FASnI_3_/C60/BCP/Ag as component materials. Another study by Chen et al. [22] enhanced the stability and performance of FASnI_3_ films using a poly(ethylene-co-vinyl acetate) antisolvent during the deposition process, achieving a PCE of 7.72% and retaining about 62.4% of its efficiency after 48 h at ambient temperatures [23,24]. A recently reported efficiency of 11.4% by adding phenyl-hydrazine hydrochloride (PHCl) to FASnI_3_ films to prevent the formation of Sn^4+^ ions and thus reduce device degradation, thereby preserving 100% of the device’s original PCE, even after 110 days in a glove box [25]. There is a promising future for stable, lead-free perovskite solar cells based on these advances. Additionally, computational studies have explored other innovative approaches for tin-based perovskites. Another simulation study conducted by Abdelaziz et al., using SCAPS-1D software, demonstrated a 14.03% efficiency using the FASnI_3_ of the active layer, TiO_2_ of the electron transport layer (ETL), and spiro-OMeTAD of the hole transport layer (HTL) [26]. This suggests that further research and development of tin-based PSCs could lead to even higher efficiencies. Additionally, these simulations provide essential insights into the most effective materials and structures for perovskite solar cells. In this context, tin-based perovskites, particularly formamidinium tin iodide (FASnI_3_), are noteworthy. They feature intrinsically favorable optical band gaps of about 1.41 eV and a high absorption coefficient, which results in a short carrier diffusion length, long carrier lifetime, low exciton binding energy, and stable electronic band structures [27,28]. These properties position FASnI_3_ as a prime candidate for further advancement in photovoltaic (PV) technology, utilizing its unique characteristics for improved solar cell performance.

Various hole transport layer (HTL) materials have been employed in conjunction with the FASnI_3_ perovskite absorber layer to enhance the performance of PSCs. Additionally, numerous electron transport layer (ETL) materials like ZnO, TiO_2_, CdS, and CeO_2_ have been utilized to improve the output performance of PSCs by facilitating the transport of electrons and holes within the device [29,30]. However, fabricating defect-free multi-layered PV devices remains a challenge, and interface defects at the ETL or HTL/perovskite interface can lead to carrier recombination and degrade overall solar cell stability. In response to these challenges, a novel concept of HTL-free solar device configuration has been recently introduced to the PV community [31,32,33,34,35]. This innovative approach aims to reduce fabrication costs and minimize interface defects without compromising cell performance. In addition to having good carrier transport, the perovskite solar cell also has a small Schottky barrier for carrier collection. Furthermore, this solar cell is made using a low-cost solution process at low temperatures. Given the substantial progress in perovskite solar cells, our research efforts have recently turned towards developing HTL-free FASnI_3_ perovskite solar cells. Experimental studies on HTL-free perovskite solar cells (PSCs) have demonstrated their potential for achieving high efficiency and improved stability [36]. For instance, fully printed methylammonium lead iodide (MAPbI3) HTL-free PSCs with carbon electrodes have shown power conversion efficiencies (PCEs) of up to 14.17% under laboratory conditions, with simplified fabrication processes, like doctor blading and methylamine vapor treatment, which are scalable and cost-effective [37]. While these studies primarily focus on lead-based perovskites, our research extends these findings to lead-free perovskite materials, such as FASnI_3_, highlighting their potential for experimental implementation in HTL-free configurations, aiming for environmentally friendly and stable solar cell technologies.

The use of the fullerene derivative C60 as an electron transport layer (ETL) has attracted significant attention in recent studies of perovskite-based solar cells. C60 offers a compelling alternative to traditional ETL materials, like TiO_2_, primarily due to its unique electronic properties and advantageous characteristics. One of the key advantages of incorporating C60 lies in its high electron mobility (~50 cm^2^/V.s), which facilitates the efficient transport of electrons generated within the perovskite absorber layer towards the external circuit [38,39]. Moreover, C60 possesses a relatively low unoccupied molecular orbital (LUMO) energy level, making it well-suited for efficient electron extraction and transport [40,41]. Additionally, the compatibility of C60 with solution processing techniques allows for facile integration into the device fabrication process, enabling cost-effective and scalable production of perovskite solar cells (PSCs). Furthermore, C60 exhibits excellent stability under operating conditions, contributing to the long-term reliability and durability of PSCs. Significant efforts have been dedicated to developing carbon-based HTL-free perovskite solar cells due to their simplified processing steps, excellent stability, and low fabrication costs. The first C60-PSCs reported in 2013 achieved an efficiency of approximately 6.6% [42]. In 2014, a study demonstrated a C60-PSC with impressive long-term stability and 12.8% efficiency [43]. Yang et al. advanced the field using C60 as the electron transport layer to create an all-carbon-based PSC with the structure FTO/C60/CH_3_NH_3_PbI_3_/carbon, achieving a notable efficiency of 15.38% [44]. Despite these advancements, the highest reported efficiency for C60-PSCs remains below 18% [33,41,45,46,47], which is still behind that of conventional PSCs with HTL. Therefore, innovative strategies are necessary to further enhance the performance of C60-PSCs. Overall, the adoption of C60 as an ETL in perovskite solar cell architectures holds great promise in enhancing device performance, scalability, and stability, thereby accelerating the commercialization and large-scale integration of this next-generation PV technology.

The main objective of this study is to investigate the performance and behavior of toxic-free FASnI_3_-based PCSs in an HTL-free configuration. Specifically, we aim to explore the use of HC(NH_2_)_2_SnI_3_ as the absorber layer and C60 as the electron transport layer (ETL) in a novel HTL-free architecture consisting of Ag/FASnI_3_/C60/FTO. This configuration will be compared to a reference structure (with HTL), Ag/PEDOT:PSS/FASnI_3_/C60/FTO, to evaluate the potential of HTL-free solar cells in achieving efficient and stable photovoltaic performance. To gain insight into device performance optimization, this study will examine several physical parameters, including active layer thickness, bulk and interface defects, ETL thickness, operating temperature, carrier concentration, and back-contact materials, to understand their impact on the key performance metrics of the devices. We have developed efficient devices by substituting the traditional TiO_2_ compact layer with fullerene C60 in a regular FASnI_3_-based PSC architecture. Additionally, we aim to demonstrate that an n-type organic charge collection layer can improve electron extraction and transport, reduce interfacial recombination, and decrease both the complexity and cost of conventional PSC designs, while also enhancing long-term performance.

## 2. Device Structure and Simulation Methodology

This study utilized SCAPS-1D, a robust simulation tool developed by the Department of Electronics and Information Systems (ELIS) at Ghent University in Belgium [48,49]. SCAPS-1D is renowned for its capability to simulate the electrical and optical properties, performance, and spectral responses of various types of solar cells. The materials studied using SCAPS-1D include perovskites, copper zinc tin sulfide (CZTS), silicon (Si), cadmium telluride (CdTe), and copper indium gallium selenide (CIGS) [50,51,52,53,54,55,56]. One of the primary benefits of using SCAPS-1D is its ability to generate simulation results that closely match experimental data, making it a reliable tool for predicting solar cell behavior. SCAPS-1D operates by solving Poisson’s equation and the continuity equations for electrons and holes. This enables accurate modeling of the dynamics and interactions of free electrons and holes within the conduction and valence bands. The software’s capabilities extend to calculating and visualizing critical electrical properties and parameters that are essential for evaluating solar cell performance. These properties include the current density–voltage (J-V) curve, which shows the relationship between the current produced by the cell and the applied voltage; the energy band structure of the heterojunction, which provides insight into the electronic properties of the material interfaces; and quantum efficiency (QE), which measures the efficiency of photon-to-electron conversion as a function of incident light wavelengths.

Additionally, SCAPS-1D can compute several critical performance metrics for solar cells. These include the open-circuit voltage (Voc), which is the maximum voltage the solar cell can produce when there is no external load; the short-circuit current (Jsc), representing the current flowing through the cell when the voltage is zero; the power conversion efficiency (PCE), which measures the percentage of solar energy converted into electrical energy or the ratio of the output power to the input power; and the fill factor (FF), which indicates the ratio of the maximum power output to the theoretical maximum power output of an ideal solar cell design. In this study, all simulations of perovskite solar cells (PSCs) were conducted under standard test conditions (STC). During the simulation, a temperature of 300 K, a light intensity of 1000 (W/m^2^), and an air mass of 1.5 AM were used. With these standardized conditions, we are able to compare simulation results with real-world performance metrics and reliably predict the behavior of solar cells under typical operating conditions [52,53]. This allows us to better understand the key factors that determine solar cell performance and design better-optimized solar cells for different conditions. Furthermore, it allows us to accurately measure the performance of solar cells.

**Figure 1:**
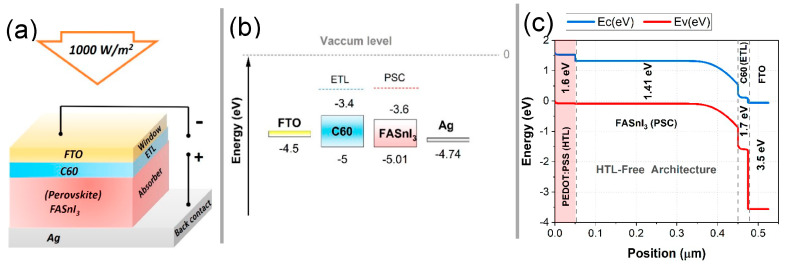
Detailed schematic of the proposed HTL-free FASnI_3_ PSC with an Ag/FASnI_3_/C60/FTO configuration: (**a**) layer structure, (**b**) band alignment diagram, and (**c**) spatial variations in conduction and valence bands, along with the energy gap.

This study aims to identify the optimum performance of a hole transport layer (HTL)-free PSC using FASnI_3_ as the active layer, which is paired with C60 as the electron transport layer (ETL). PV key performance parameters were evaluated using SCAPS-1D algorithms. For the reference structure (Ag/PEDOT:PSS(HTL)/FASnI_3_(PSC)/C60(ETL)/FTO), the FASnI_3_ layer thickness was initially fixed at 450 nm, as depicted in Figure 1. This study explored variations in the absorber and ETL thicknesses, bias voltages, and doping concentrations to optimize the device. In addition to providing insights into the impact on the varied fields, the optimization process included insights into the impact on absorbers and ETLs, bias voltages, bulk defects, carrier concentrations within ETLs, operating temperatures, back-contact materials, and doping concentrations, as indicated in Table 1 and Table 2. In Table 1, we present the essential physical parameters utilized in the simulation of the proposed HTL-free FASnI_3_ structure. Interfacial properties and contacts play a significant role in the efficiency of PSC devices. Based on the reference model, Table 2 illustrates the parameters used to model interface defects at the HTL/FASnI_3_ and FASnI_3_/ETL interfaces of perovskites. This comprehensive simulation approach ensures an accurate representation of device behavior, facilitating the optimization of device parameters. This simulation provides insight into the physical mechanisms governing charge transport and recombination. By utilizing this knowledge, we will be able to enhance and improve tin-based photovoltaic devices, ultimately leading to a more efficient and stable device.

## 3. Results and Discussion

### 3.1. Impact of the HTL Layer on FASnI_3_-PSC Performance

**Figure 2:**
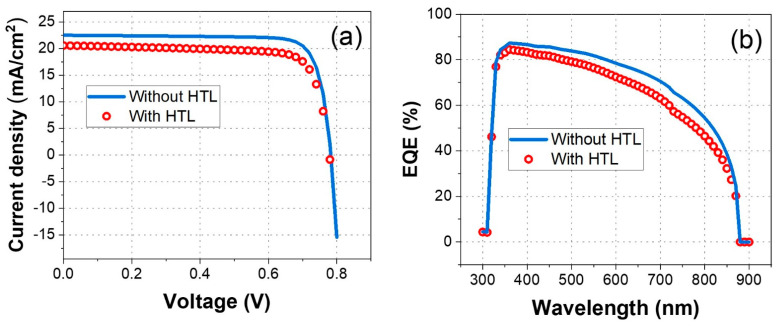
(**a**) J-V characteristics of FASnI_3_ PSCs without the HTL (blue solid line) and with the HTL (red circles). (**b**) The EQE of FASnI_3_ PSCs without the HTL (blue line) and with the HTL (red circles).

This study was initiated to investigate the impact of the hole transport layer on the performance of formamidinium tin iodide perovskite solar cells (PSCs). To address this, current density versus voltage (J-V) characteristics and the external quantum efficiency (EQE) were analyzed for PSCs with Ag/PEDOT:PSS(HTL)/FASnI_3_ (PSC)/C60(ETL)/FTO and without an HTL (Ag/FASnI_3_ (PSC)/C60(ETL)/FTO), as shown in Figure 2. The comparison aims to understand how the presence or absence of the HTL affects key photovoltaic parameters such as the short-circuit current density (JSC), open-circuit voltage (VOC), and fill factor (FF). The results of these analyses are presented in Table 3, highlighting the performance variations between the two configurations.

Figure 2 illustrates the current density versus voltage (J-V) characteristics (Figure 2a) and the external quantum efficiency (EQE) (Figure 2b) of formamidinium tin iodide (FASnI_3_) perovskite solar cells (PSCs) with and without an HTL. Figure 2a shows that the PSC without the HTL (depicted by a solid blue line) achieves a higher short-circuit current density compared to the PSC with the HTL (represented by red circles), suggesting better charge extraction and minimized recombination losses in the HTL-free configuration. The open-circuit voltage is also slightly higher for the HTL-free device, indicating more efficient charge separation and reduced voltage loss. In Figure 2b, the HTL-free PSC (solid blue line) exhibits higher efficiency over a wide range of wavelengths (350–850) nm compared to the PSC with the HTL (red circles). The peak EQE is higher in the HTL-free configuration, suggesting improved photon absorption and charge carrier generation efficiency. The broader and higher EQE response in the HTL-free PSC highlights its superior performance in converting incident light into electrical current across the solar spectrum.

Table 3 highlights a significant improvement in FASnI_3_ PSC photovoltaic performance parameters when the hole transport layer (HTL) is omitted. Specifically, power conversion efficiency (PCE) increases from 12.53% with the HTL to 14.46% without the HTL.

### 3.2. Impact of Bulk and Interface Defect Density on HTL-Free PSC PV Performance

**Figure 3:**
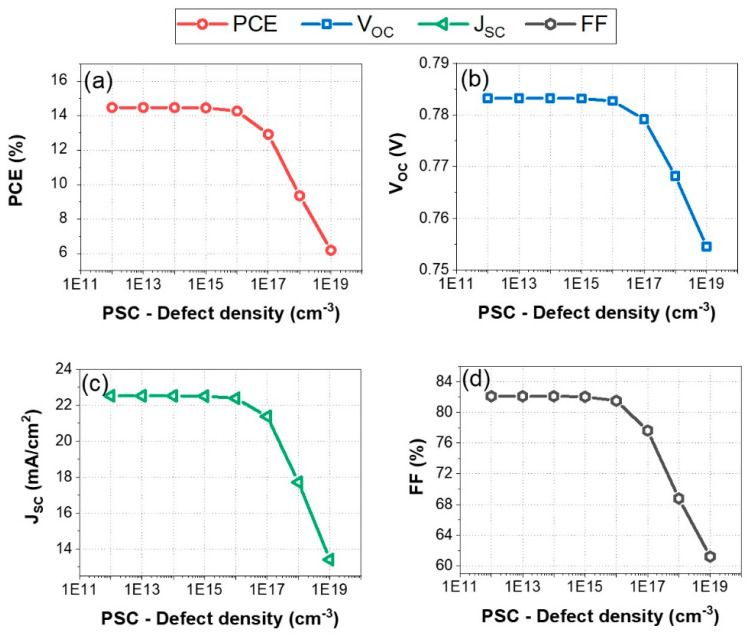
Impact of bulk defect density on the PV performance metrics of the HTL-free FASnI_3_ structure: (**a**) PCE, (**b**) *VOC*, (**c**) *JSC*, and (**d**) FF.

The performance of perovskite solar cells (PSCs) is highly sensitive to the bulk defect density because defects in the material can act as recombination centers for charge carriers (electrons and holes). The bulk defect density in the active layer of the device, as shown in Figure 3, varied over a range from 10^11^ cm^−3^ to 10^19^ cm^−3^, illustrating its impact on the performance metrics of perovskite solar cells.

This broad range allows for a comprehensive analysis of how increasing defect density affects the PV performance of the HTL-free FaSnI_3_ cell design. According to Figure 3, the power conversion efficiency (PCE) remains relatively stable at approximately 15–16% for defect densities ranging from 10^11^ cm^−3^ to 10^15^ cm^−3^. Beyond 10^15^ cm^−3^, there is a sharp decline in the PCE, dropping to around 6% at 10^19^ cm^−3^ (Figure 3a). Similarly, the open-circuit voltage (V_OC_) is fairly stable at around 0.78 V for defect densities up to 10^15^ cm^−3^ (Figure 3b), but it decreases significantly beyond this point, reaching about 0.75 V at 10^19^ cm^−3^ (Figure 3b). The short-circuit current density remains consistent at approximately 24 mA/cm^2^ up to 10^15^ cm^−3^, after which it decreases sharply to around 14.3 mA/cm^2^ at 10^19^ cm^−3^, as shown in Figure 3c. The fill factor (FF) also remains high, around 82–84%, for defect densities of up to 10^15^ cm^−3^, but it shows a marked decline beyond this threshold, falling to approximately 60% at 10^19^ cm^−3^, as depicted in Figure 3d. These findings indicate that the PSC photovoltaic performance is significantly affected by the bulk defect density, with all metrics (PCE, V_OC_, J_SC_, and FF) displaying threshold behavior where they remain relatively stable up to around 10^15^ cm^−3^ and then degrade rapidly as the defect density increases further.

The results of this study indicate that the HTL-free device is capable of tolerating defects up to a certain density of ~10^15^ cm^−3^ without significantly hindering charge transport and recombination. Beyond this threshold, defects become numerous enough to dominate the recombination processes, leading to rapid performance degradation. Below this threshold, charge carriers can travel longer distances before recombining, meaning that more carriers contribute to the photocurrent. Above this threshold, the mean free path of carriers is significantly reduced due to frequent recombination at defect sites. Defect densities below 10^15^ cm^−3^ generally indicate good material quality and uniformity, which are crucial for high performance. Higher defect densities indicate poor crystallinity and more grain boundaries, which adversely affect all performance metrics. Therefore, keeping the defect density below 10^15^ cm^−3^ is essential to ensure minimal recombination losses, optimal charge carrier dynamics, and high-quality material properties, all of which are crucial for achieving maximum performance in perovskite solar cells.

**Figure 4:**
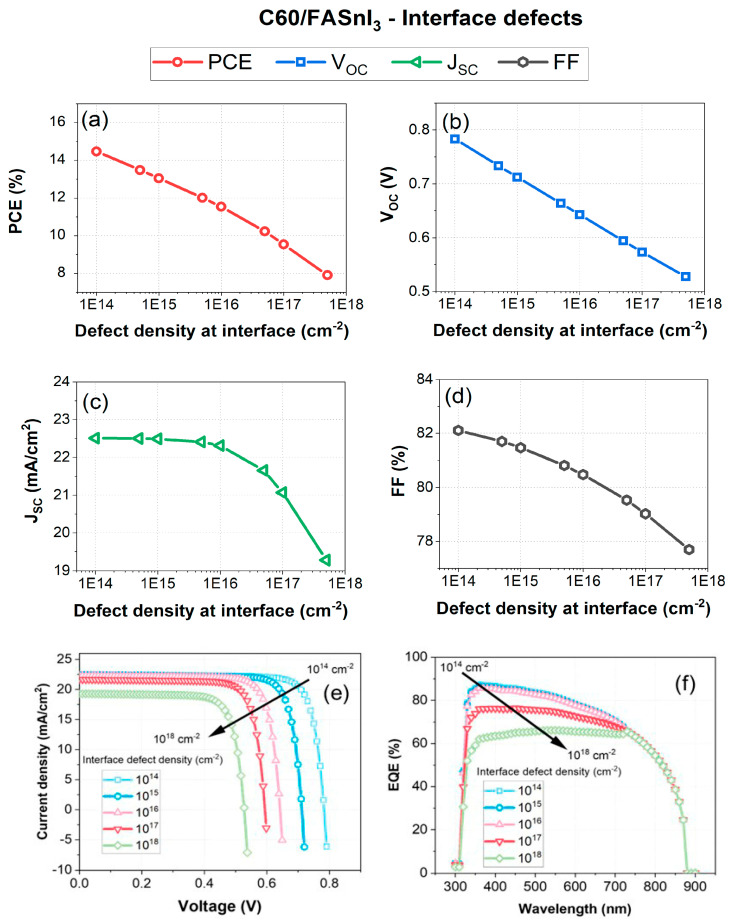
Impact of interface defect density on the PV performance parameters of the HTL-free FASnI_3_ structure: (**a**) PCE, (**b**) *V*_*OC*_, (**c**) *J*_*SC*_, (**d**) FF, (**e**) J-V characteristics, (**f**) EQE versus wavelength.

The proposed HTL-free architecture reduces the complexity and costs associated with the device by eliminating the HTL layer, thereby decreasing the number of interfaces. Additionally, this design allows for a more precise identification of interface defects, which act as non-radiative recombination centers and significantly impact the device’s performance. In general, the quality of the interfaces within the device has a significant impact on its performance. Interfaces in PSCs include junctions between different layers, such as the absorber layer and the electron transport layer (ETL). Defects at these interfaces, including vacancies, interstitials, and grain boundaries, can act as non-radiative recombination centers. These defects trap charge carriers and facilitate recombination processes, thereby reducing charge carrier collection efficiency and overall device performance. Figure 4 illustrates the impact of interface defect density at the ETL/PSC (C60/FASnI_3_) interface on various performance metrics of the HTL-free FASnI_3_ structure. As depicted in Figure 4a, the power conversion efficiency decreases from approximately 15% to 7% as the defect density increases from 1 × 10^14^ cm^−2^ to 1 × 10^18 ^cm^−2^. This decrease in the PCE is attributed to the higher recombination rates introduced by increased defect densities, which hinder the efficient collection of charge carriers at the back and front of the device.

The recombination losses can be modeled using the Shockley–Read–Hall (SRH) recombination theory as expressed by Equation (1) [54] as follows:

(1) $$R_{SRH}=\frac{np-n_{i}^{2}}{\tau_{p}\left(n+n_{1}\right)+\tau_{n}\left(p+p_{1}\right)}$$

where *R*SRH is the recombination rate, *n* and *p* are the electron and hole densities, *ni* is the intrinsic carrier concentration, and *τ*n and *τp* are the electron and hole lifetimes, respectively, which are inversely related to the defect density [55]. According to Shockley–Read–Hall (SRH), the rate of electron and hole recombination is proportional to the product of their concentrations and inversely proportional to the electric field. The presence of interface defects such as vacancies, interstitials, and grain boundaries can result in an increased recombination of charge carriers, thereby reducing the efficiency of the device.

Figure 4b depicts the relationship between the open-circuit voltage (*V*OC) and defect density. The *V*OC decreases linearly with increasing defect density, falling from 0.77 V at 10^14^ cm^−2^ to 0.55 V at 1018 cm^−2^. This 28% reduction in the *V*OC can be explained by the increased presence of recombination centers due to defects, which lower the effective built-in potential of the solar cell. Similarly, Figure 4c shows that the short-circuit current density (*J*SC) drops from 23 mA/cm^2^ to approximately 19.5 mA/cm^2^, representing a 15% decrease, likely due to defects trapping charge carriers and reducing collection efficiency. This behavior is consistent with increased recombination losses observed with higher defect densities. The reduction in the JSC is explained by the trapping of charge carriers at defect sites, which diminishes the current generated by photogenerated carriers. Accordingly, the fill factor (FF), as shown in Figure 4d, also decreases with increasing defect density, declining from 82% to 77% as a result of increased recombination losses and increased shunt resistance. The decline in the FF is due to increased series resistance (*Rs*) and decreased shunt resistance (*Rsh*), which are caused by higher defect densities and negatively impact charge carrier extraction and overall solar cell performance, as described in Equation (2). The shunt resistance in relation to defect density is given by (*Rsh* ∝ 1/N_D), where *ND* is the defect density [56].

(2) $$FF=\frac{V_{MP}\times J_{MP}}{V_{OC}\times J_{SC}}$$

where *V*_MP_ and *J*_MP_ are the voltage and current at the maximum power point, respectively. Both *V*_MP_ and *J*_MP_ are negatively affected by higher defect densities [57]. Figure 4e,f provide further insights into the impact of defect density on the J-V characteristics and the EQE. In Figure 4e, the J-V curves show that higher defect densities result in a more rapid decrease in current density at higher voltages. This behavior is consistent with increased recombination losses observed with higher defect densities. This phenomenon is explained by the diode equation (Equation (3)), which indicates that higher J_0_ values, indicative of increased recombination, cause a reduction in current when the applied voltage is raised.

(3) $$J=J_{SC}-J_{0}\left(e^{\frac{qV}{nkT}}-1\right)$$

where *J*_0_ is the reverse saturation current density, *n* is the ideality factor, *q* is the elementary charge, *k* is the Boltzmann constant, and *T* is the temperature [58]. As a result of the optical EQE spectra response to the interface defect density, the external quantum efficiency (EQE) significantly decreases across all wavelengths from 300 to 700 nm. Figure 4f demonstrates that the EQE decreases significantly with higher defect densities across the spectrum, with the peak EQE dropping from about 85% at 10^14^ cm^−2^ to around 40% at 10^18^ cm^−2^, indicating more than a 50% reduction. This reduction is due to increased recombination within the absorber layer, as higher defect densities create more recombination centers, reducing the number of photogenerated carriers collected. This decrease in the EQE suggests that higher defect densities lead to lower charge collection efficiency due to enhanced recombination at defect sites, as described in Equations (4) and (5). Accordingly, higher interfacial defect densities reduce the number of collected charge carriers, thereby decreasing the EQE.

(4) $$EQE(\lambda )=\frac{\;\mathrm{number}\;\mathrm{of}\;\mathrm{collected}\;\mathrm{charge}\;\mathrm{carriers}\;}{\;\mathrm{number}\;\mathrm{of}\;\mathrm{incident}\;\mathrm{photons}\;}$$

(5) $$EQE(\lambda )=\frac{\eta_{\mathrm{col}\;}(\lambda )\eta_{\mathrm{ext}\;}(\lambda )\eta_{\mathrm{itt}\;}(\lambda )}{1+N_{D}\cdot f(\lambda )}$$

where *η*_col_, *η*_ext_, and *η*_int_ are the collection, extraction, and internal quantum efficiencies, respectively, and *f*(*λ*) is a factor related to defect density [59]. In the C60/FASnI_3_ PV configuration, the performance parameters are significantly affected by the density of the interface defects. A higher defect density results in higher recombination rates, which degrade key performance parameters, such as the PCE, V_OC_, J_SC_, and FF. Optimum performance and efficiency of the HTL-free PSC are dependent on minimizing interface defect density. Therefore, to achieve optimal device performance, it is crucial to minimize defect density below 1 × 10^15^ cm^−3^ through high-quality material synthesis, effective defect passivation techniques, and careful interface engineering.

### 3.3. Impact of Absorber Thickness on HTL-Free PSC PV Performance

**Figure 5:**
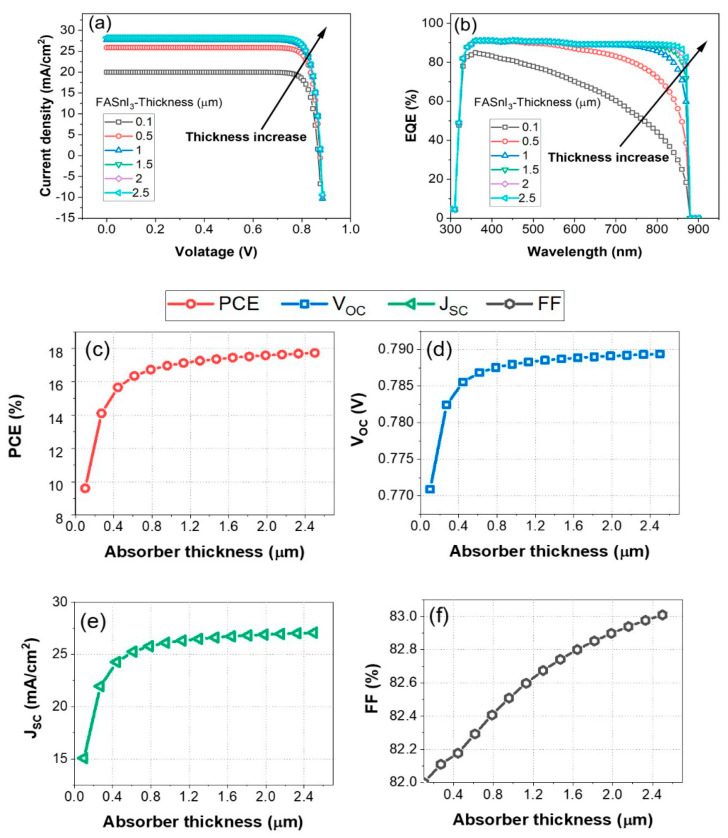
The impact of active layer (absorber) thickness (FASnI_3_) ranging from 10 nm to 2500 nm on the PV performance parameters of the HTL-free FASnI_3_ structure: (**a**) PCE, (**b**) *VOC*, (**c**) *JSC*, (**d**) FF.

This section explores the critical role of the active layer (absorber) in determining the performance on HTL-free device architecture. For optimal performance in an HTL-free configuration, the active layer must exhibit several key characteristics. It should possess high light absorption to maximize photon capture, efficient charge carrier generation to ensure a high density of free electrons and holes, and excellent charge transport properties to facilitate the swift movement of these carriers to the respective electrodes. Additionally, the active layer should demonstrate robust stability and compatibility with other device components, ensuring long-term reliability and sustained functionality of the solar cell. These attributes collectively contribute to achieving high efficiency and durability in HTL-free perovskite solar cells. Figure 5a–f illustrate the impact of absorber thickness, ranging from 100 nm to 2500 nm, on various performance metrics of the C60/FASnI_3_ solar cell. These measures include J-V characteristics, the EQE, PCE, V_OC_, J_SC_, and FF.

According to Figure 5a, increasing the absorber thickness from 0.1 µm to 2.5 µm results in enhanced current density, which is particularly noticeable at higher voltages. Thicker absorbers facilitate higher current densities due to increased photon absorption and charge carrier generation. For instance, at 0.8 V, the current density for 0.1 µm is about 18 mA/cm^2^, while for 2.5 µm, it reaches approximately 27 mA/cm^2^. This can be attributed to the diode equation (Equation (3)). Thicker absorbers enhance light absorption across a broader spectrum, resulting in more efficient generation and collection of charge carriers. For example, at 800 nm, the EQE increases from around 40% for 0.1 µm to about 90% for 2.5 µm, as shown in Figure 5b. As the thickness increases, more photons are absorbed, generating more electron–hole pairs, thereby increasing the EQE. As illustrated in Figure 5c, the PCE increases due to thicker absorbers that generate and collect charge carriers more efficiently. Thicker absorbers generate more charge carriers due to enhanced light absorption. The short-circuit current density (JSC) can be expressed by Equation (6) as follows:

(6) $$J_{SC}=q\int_{0}^{\lambda_{g}}\varphi (\lambda )EQE(\lambda )d\lambda$$

where Φ(*λ*) is the photon flux and the EQE (*λ*) is the external quantum efficiency [60].

Before saturation occurs, the PCE increases from approximately 12% at 100 nm to approximately 17.6 % at 1500 nm. This can be explained by the fact that most of the incident photons are absorbed within a certain depth of the absorber layer, known as the absorption depth, which is dependent on the material’s absorption coefficient and the wavelength of light. As the absorber thickness increases, the path length that charge carriers must travel to reach the electrodes also increases. In thicker layers, the likelihood of recombination events occurring before the carriers are collected is higher, particularly for charge carriers generated deep within the absorber layer. This is particularly true when the carriers are generated deep within the active layer. As a response to this increased recombination, carrier collection is reduced, leading to a plateau or saturation in the overall performance of the PV device.

As shown in Figure 5d, the V_OC_ increases from approximately 0.775 V at 0.1 µm to around 0.790 V at 1.5 µm, and then stabilizes with further thickness increases. The series resistance of the solar cell increases as the absorbing layer becomes thicker. In this case, the carriers have to travel a greater distance through the material, encountering more resistance along the way. An increase in series resistance reduces the fill factor (FF) and the voltage across the open circuit (V_OC_). This indicates that enhanced charge carrier collection and reduced recombination with increased thickness contribute to a higher V_OC_. As can be seen in Figure 5e, the J_SC_ increases from approximately 15 mA/cm^2^ to approximately 27 mA/cm^2^ at 1.5 m, after which it reaches saturation. This trend reflects the improved photon absorption and charge carrier generation with thicker absorbers up to an optimal thickness of about 1500 nm, as described by Equation (6), beyond which additional thickness does not significantly increase the J_SC_ due to the saturation effect. According to Figure 5f, the FF increases continuously from approximately 82% at 100 nm to approximately 83% at 2500 nm, indicating enhanced electrical properties and reduced recombination rates. This gradual improvement in the FF highlights the overall enhancement in device performance with optimized absorber thickness.

This study has demonstrated that there is an optimal range of active layer thickness for C60/FASnI_3_ solar cells. The cells exhibit peak performance characteristics when the absorber thickness is between 1.5 µm and 2 µm. Within this range, the power conversion efficiency reaches approximately 17%, the open-circuit voltage stabilizes around 0.785 V, and the short-circuit current density peaks at about 27 mA/cm^2^. Additionally, the fill factor improves to around 83% with increased thickness. These results indicate that within this optimal thickness range, the absorber layer effectively balances photon absorption, charge carrier generation, and electrical properties, leading to maximum device efficiency. Beyond this range, further increases in thickness yield diminishing returns due to enhanced recombination and resistive losses. To achieve the highest performance in HTL-free C60/FASnI_3_ solar cells, it is crucial to maintain an absorber layer thickness between 1.5 µm and 2 µm.

### 3.4. Impact of ETL (C60) Thickness and Doping on HTL-Free PSC PV Performance

**Figure 6:**
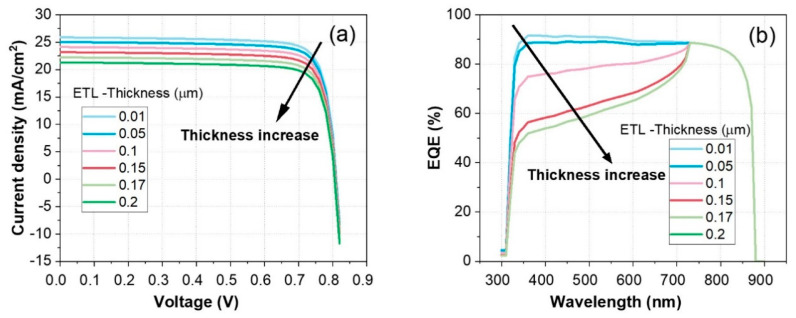
Impact of ETL layer thickness (C60) ranging from 10 nm to 200 nm on the PV performance of the HTL-free FASnI_3_ structure: (**a**) current density–voltage (J-V) characteristics, (**b**) external quantum efficiency (EQE) versus wavelength.

A thorough understanding of the effects of the ETL thickness on the performance of C60/FASnI_3_ solar cells is essential to optimizing the efficiency of HTL-free devices. For superior performance of solar cells, the ETL thickness must be maintained at an optimal value, as demonstrated in this study. Increasing the thickness of the ETL negatively affects both the current density and the external quantum efficiency. Figure 6 illustrates the impact of electron transport layer (ETL) thickness on the performance metrics of C60/FASnI_3_ solar cells, focusing on current density–voltage and external quantum efficiency.

Figure 6a shows that as the ETL thickness increases from 10 nm to 200 nm, the current density decreases at higher voltages. For instance, at 0.8 V, the current density drops from about 27 mA/cm^2^ for an ETL thickness of 10 nm to approximately 24 mA/cm^2^ for an ETL thickness of 200 nm. This decrease in the J_SC_ can be attributed to increased series resistance and reduced charge carrier mobility in thicker ETLs, which hinder efficient charge extraction. Figure 6b further supports these findings by demonstrating how the ETL thickness affects the EQE at different wavelengths.

Figure 5b demonstrates the effect of the ETL thickness on the EQE across different wavelengths. As the ETL thickness increases, the EQE decreases significantly, especially in the longer wavelength range. For example, at 700 nm, the EQE drops from around 90% for an ETL thickness of 0.01 µm to about 40% for an ETL thickness of 0.2 µm. This reduction is due to the increased recombination losses and reduced light absorption efficiency in thicker ETLs, which limit the generation and collection of photogenerated carriers.

**Figure 7:**
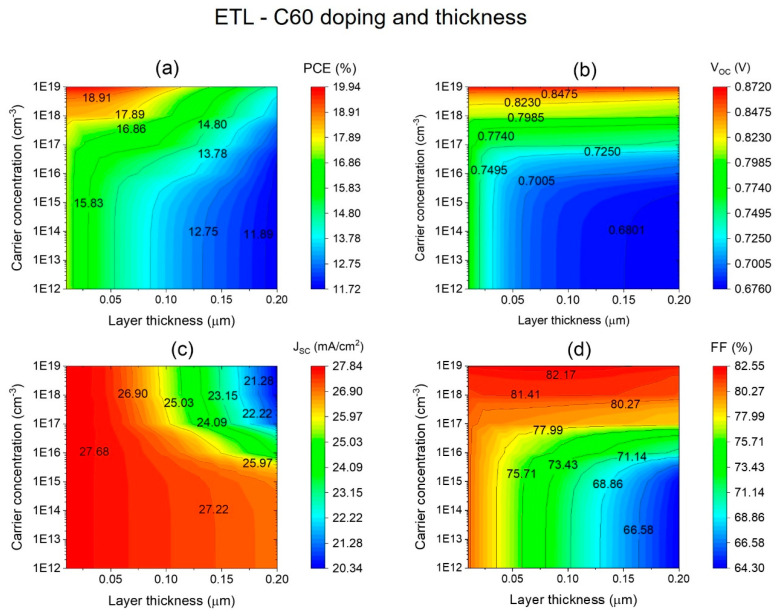
Analysis of the effects of the ETL (C60) thickness in the range of 10–200 nm and doping level from 1012 to 1019 cm^−3^ on key PV performance metrics of the HTL-free FASnI_3_ structure: (**a**) PCE, (**b**) *VOC*, (**c**) *JSC*, (**d**) FF.

Overall, the results indicate that maintaining a thinner ETL is crucial for optimizing the performance of C60/FASnI_3_ solar cells, balancing both efficient charge transport and high light absorption. This study reveals that increasing the ETL thickness adversely affects both the current density and the external quantum efficiency (EQE), highlighting the importance of maintaining an optimal ETL thickness for superior solar cell performance. To identify the optimal thickness and carrier concentration for the C60 electron transport layer (ETL), a detailed correlation study was conducted to examine how these parameters influence device performance. The doping level of the C60-ETL was adjusted systematically from 10^12^ cm^−3^ to 10^19^ cm^−3^, while the layer’s thickness varied from 10 nm to 200 nm, as illustrated in Figure 7. Figure 7a–d depict the combined effects of the ETL thickness and carrier concentration on the performance metrics of HTL-free C60/FASnI_3_ solar cells, including the PCE, V_OC_, J_SC_, and FF.

PV performance metrics are illustrated in Figure 7a–d based on carrier concentration (*y*-axis) and C60 thickness (*x*-axis). The results indicate a significant decline in device performance as the C60-ETL thickness increases from 10 nm to 200 nm. Specifically, Figure 7a demonstrates a reduction in power conversion efficiency from 18.91% to 11.89% as the thickness increases from 10 nm to 200 nm at a carrier concentration of 1019 cm^−3^. Furthermore, Figure 7b reveals that decreasing the ETL thickness from 200 nm to 50 nm at a high carrier concentration of 10^19^ cm^−3^ enhances the *V*_*OC*_ from 0.680 V to 0.872 V. For the *JSC* and FF, as shown in Figure 7c,d, both parameters show considerable improvements with decreasing C60-ETL thickness and increasing carrier concentration. Figure 7c highlights that the *J*_*SC*_ rises from 20.34 mA/cm^2^ to 27.68 mA/cm^2^ as the ETL thickness is reduced from 200 nm to 50 nm at a carrier concentration of 10^19^ cm^−3^. Similarly, Figure 7d illustrates that the fill factor (FF) improves from 66.58% to 82.17% under the same conditions. These results indicate that optimizing the C60-ETL thickness to around 50 nm, in conjunction with a high carrier concentration of 10^19^ cm^−3^, is crucial for achieving maximum performance in C60/FASnI_3_ solar cells. The findings obtained in this study demonstrated that the highest PCE of 18.91% was achieved with an ETL thickness of 50 nm and a carrier concentration of 10^19^ cm^−3^. As a result, the open-circuit voltage peaked at 0.872 V, the short-circuit current density reached 27.68 mA/cm^2^, and the fill factor attained 82.17% under these optimal conditions. Increasing the ETL thickness beyond 150 nm led to significant declines in all key performance parameters, highlighting the critical balance required between the ETL thickness and carrier concentration to maximize solar cell efficiency.

### 3.5. Impact of Operating Temperature on HTL-Free PSC PV Performance

**Figure 8:**
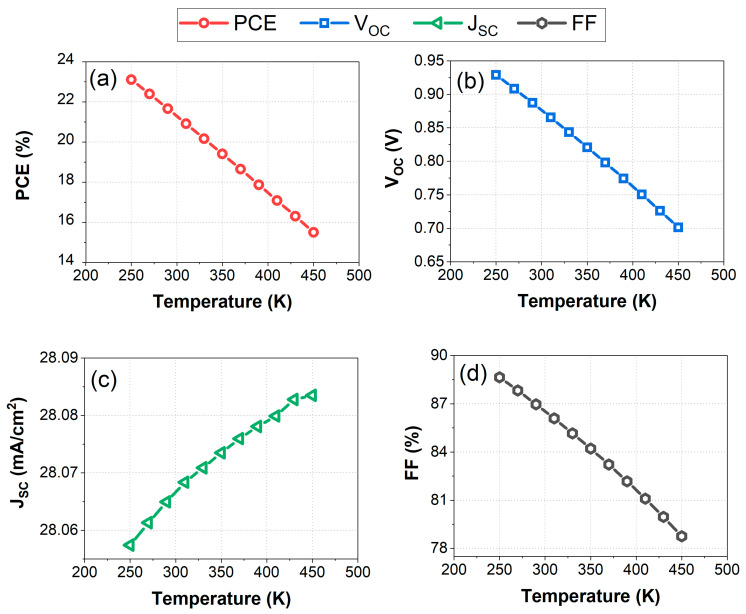
Temperature-dependent photovoltaic metrics for the HTL-free FASnI_3_ structure (250 K to 450 K): (**a**) PCE, (**b**) *VOC*, (**c**) *JSC*, (**d**) FF.

The objective of this study is to investigate the influence of temperature on the structure of the HTL-free structure composed of FTO/C60/FASnI_3_/Ag in the temperature range of 275 K to 450 K. In Figure 8a–d, we illustrate the effects of operating temperature on key photovoltaic performance parameters. The chosen temperature range of 275 K to 450 K is significant as it covers a wide range of operating conditions, allowing for a comprehensive understanding of how temperature affects the performance of the HTL-free structure. This range encompasses both typical operating temperatures and extreme conditions, providing valuable insights into the stability and efficiency of the photovoltaic system across various thermal environments. The operating temperature significantly impacts the performance metrics of the FTO/C60/FASnI_3_/Ag perovskite solar cell (PSC). As shown in Figure 8a–d, increasing the temperature from 250 K to 450 K results in notable declines in key performance parameters. The power conversion efficiency (PCE) drops from approximately 21.95% at 250 K to about 14% at 450 K, reflecting the overall degradation in device performance due to higher temperatures. The open-circuit voltage (VOC) decreases linearly from around 0.90 V at 250 K to approximately 0.70 V at 450 K, indicating increased recombination rates and reduced effective carrier lifetimes at elevated temperatures. The reduction in the VOC with increasing temperature is due to the increased intrinsic carrier concentration, which enhances recombination. The relationship between the VOC and temperature is given by Equation (7) as follows:

(7) $$V_{OC}=\frac{kT}{q}ln\left(\frac{J_{SC}}{J_{0}}+1\right)$$

As temperature (*T*) increases, the term *kT*/q increases, leading to a reduction in the VOC if other factors remain constant [61]. Furthermore, the reverse saturation current density (*J*0) typically increases exponentially with temperature due to increased carrier recombination [62]. This increase in *J*_0_ results in a lower value of the logarithmic term, further reducing the VOC, as observed in Figure 8b.

In contrast, the short-circuit current density shows a slight increase from about 28.06 mA/cm^2^ at 250 K to around 28.09 mA/cm^2^ at 450 K. This minor change suggests that the thermal generation of charge carriers marginally enhances the JSC, but not enough to counteract the overall performance decline. The fill factor (FF) experiences a significant reduction, dropping from around 87% at 250 K to about 78% at 450 K. This decrease in the FF is primarily due to increased series resistance and decreased shunt resistance, which adversely affect the electrical properties of the solar cell. Overall, the analysis highlights that while the FTO/C60/FASnI_3_/Ag PSC structure can maintain stable performance up to 340 K, higher temperatures lead to substantial efficiency losses, emphasizing the need for effective thermal management to sustain optimal device performance.

Based on the provided data, the performance metrics (PCE, VOC, JSC, and FF) degrade significantly as the temperature increases beyond 300 K. For practical and efficient operation, the temperature stability range should ideally be between 250 K and 340 K. Within this range, the PCE remains relatively high, and the decreases in the VOC and FF are manageable, ensuring efficient operation of the solar cells. Operating outside this range, particularly above 340 K, leads to significant performance losses due to increased recombination rates and resistive losses. The stable performance of the FTO/C60/FASnI_3_/Ag perovskite solar cell (PSC) structure is maintained up to an operating temperature of 340 K. At this temperature, the device exhibits reliable performance with a power conversion efficiency (PCE) of approximately 19%, an open-circuit voltage of about 0.82 V, a short-circuit current density of around 28.07 mA/cm^2^, and a fill factor (FF) of approximately 84%. These values indicate that up to 340 K, the solar cell maintains a good balance between efficiency and operational stability. Although there is a decline in performance compared to lower temperatures, the solar cell still operates effectively, minimizing the adverse effects of increased recombination and resistive losses. Ensuring the operating temperature does not exceed 340 K is crucial for sustaining the balance between efficiency and long-term stability, optimizing the overall performance of the perovskite solar cell under practical conditions.

### 3.6. Impact of the Back-Contact Work Function on HTL-Free PSC PV Performance

**Figure 9:**
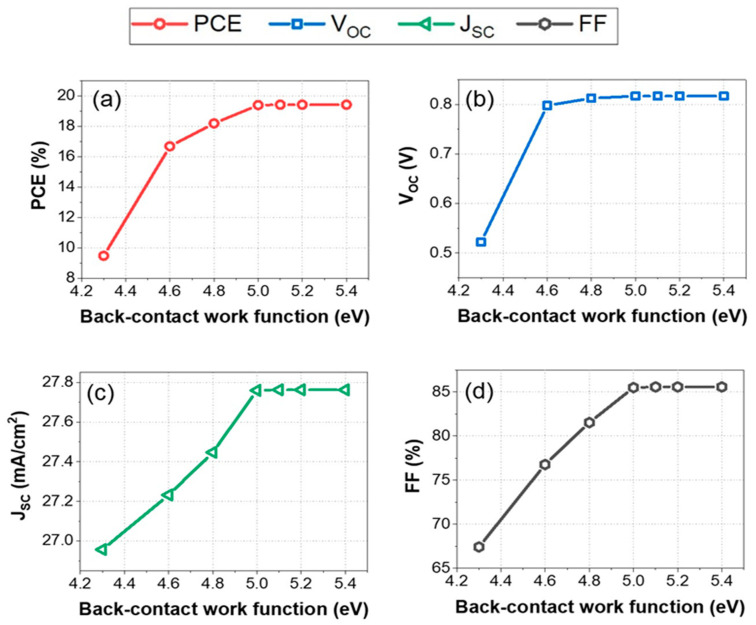
Variation of photovoltaic parameters with the back-contact work function in the range of 4.3 eV to 5.4 eV for the HTL-free FASnI_3_ structure: (**a**) power conversion efficiency (PCE), (**b**) open-circuit voltage (*V_oc_*), (**c**) short-circuit current density (Jsc), (**d**) fill factor (FF).

Figure 9 illustrates the influence of the back-contact work function on the performance metrics of HTL-free FASnI_3_ solar cells. Figure 9a depicts that the PCE increases from approximately 9.24% at a back-contact work function of 4.2 eV to approximately 19.68% at 5.4 eV. This improvement is due to better alignment of the energy levels at the interface between the back contact and the FASnI_3_ layer, which reduces charge carrier recombination and enhances carrier extraction efficiency. The open-circuit voltage increases sharply from approximately 0.53 V at 4.2 eV to around 0.81 V at 4.6 eV and then stabilizes.

This sharp increase is attributed to the reduction in interface recombination losses with improved work function alignment, leading to a higher built-in potential and better charge separation. In terms of short-circuit current density, around 27.2 mA/cm^2^ is obtained at 4.2 eV, whereas around 278 mA/cm^2^ is obtained at 5.4 eV. This increase is due to enhanced carrier collection efficiency with better energy level alignment, reducing recombination at the back contact. Furthermore, the fill factor increases from approximately 68.4% at 4.2 eV to around 86.2% at 5.4 eV. This reflects reduced series resistance and improved charge extraction efficiency. It is concluded from the analysis that optimizing the back-contact work function can significantly enhance the performance of HTL-free FASnI_3_ solar cells. A work function of 5 eV results in a PCE of approximately 19.63%, a VOC of 0.81 V, a JSC of 27.65 mA/cm^2^, and an FF of 85%. These findings indicate that optimizing the back-contact work function to about 5 eV significantly improves the performance of HTL-free FASnI_3_ solar cells, resulting in a higher PCE, V_OC_, J_SC_, and FF. Thus, it can be concluded that optimizing the back-contact work function is an important factor in increasing the performance of FASnI_3_ solar cells. This optimization is crucial for maximum device efficiency, highlighting the importance of proper energy level alignment in solar cell design.

**Figure 10:**
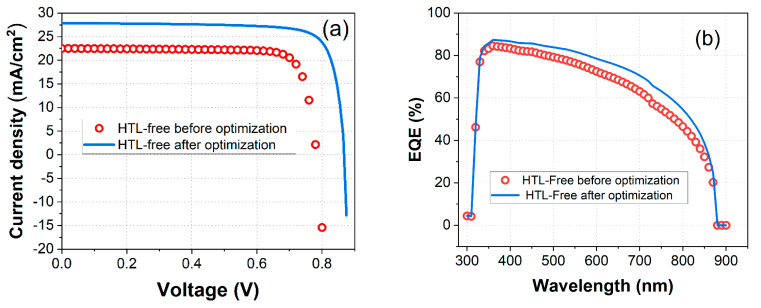
Comparison of photovoltaic parameters for the HTL-free FASnI_3_ structure before (red circles) and after optimization (solid line blue): (**a**) current density–voltage (J-V) characteristics, (**b**) external quantum efficiency (EQE).

Based on Figure 10, Table 4 summarizes the comparison between the proposed HTL-free model prior to and following optimization. Figure 10 illustrates the impact of optimization on the performance of HTL-free FASnI_3_ solar cells, comparing the current density-voltage (J-V) characteristics (Figure 10a) and external quantum efficiency (EQE) spectra (Figure 10a) before and after optimization. Before optimization, the J-V curve (red circles) showed a lower current density and a pronounced decline at higher voltages. After optimization, the J-V curve (blue line) exhibits a significant improvement in current density, maintaining a high value across the voltage range, particularly at higher voltages.

The improved J-V characteristics after optimization indicate enhanced charge carrier generation and collection efficiency. The optimization likely involved reducing recombination losses and improving the charge transport properties within the device. This results in a higher short-circuit current density and open-circuit voltage, which contribute to overall improved performance. Specifically, the J_SC_ increased from 22.50 mA/cm^2^ to 27.86 mA/cm^2^, and the V_OC_ improved from 0.78 V to 0.87 V. The power conversion efficiency (PCE) was also observed to have a substantial increase from 14.46% to 19.63%, while the fill factor (FF) remained relatively stable, slightly decreasing from 82.09% to 81%, as summarized in Table 4.

The optimization of the HTL-free FASnI_3_ PV structure leads to significant improvements in both current density–voltage (J-V) characteristics and external quantum efficiency. The optimized cells exhibit higher current density, better voltage performance, and increased EQEs from 350 to 850 nm across a wide range of wavelengths, as depicted in Figure 10b. These enhancements are attributed to reduced recombination losses, improved charge transport, and better photon absorption, resulting in overall superior device performance.

## 4. Conclusions

In conclusion, our study focused on the advanced modeling and optimization of an HTL-free FASnI_3_/C60 perovskite solar cell, aiming to enhance performance by eliminating the hole transport layer (HTL). Our efforts to eliminate the HTL have resulted in a simplified device architecture that can reduce the complexity of PSC fabrication and reduce its cost. Furthermore, the absence of the HTL resulted in improved charge extraction and reduced charge recombination, leading to higher power conversion efficiency and enhanced overall performance of the solar cell. The key parameters of solar cell performance were systematically evaluated using the solar cell capacitance simulator (SCAPS-1D). Our findings revealed that the HTL-free configuration significantly improved I-V characteristics, leading to a notable increase in power conversion efficiency. Additional performance improvements were achieved by optimizing parameters such as active layer thickness, defect concentrations, C60-ETL thickness, carrier concentration, and back-contact materials. The optimized HTL-free FASnI_3_ structure achieved a maximum PCE of 19.63%, underscoring its potential for creating highly efficient, cost-effective, and environmentally friendly photovoltaic technology solutions. As a result of this work, a significant step forward has been made in the development of advanced perovskite solar cells, paving the way for practical applications. These findings suggest that the HTL-free FASnI_3_ structure could be a viable candidate for the commercial-scale production of perovskite solar cells.

## Figures and Tables

**Table 1:** Comprehensive list of physical parameters used in both the reference and HTL-free model simulation at 300 K [8,22,23,31,32].

Parameters	ETL (C60)	Absorber (FASnI_3_)	HTL (PEDOT: PSS)
Thickness (μm)	0.025 *	0.55 *	0.05
Band gap (eV)	1.7	1.41	1.6
Electron affinity (eV)	4.5	3.62	3.4
Dielectric permittivity	6	10	3
CB effective DOS (cm^−3^)	2.2 × 10^19^	1 × 10^18^	2.2× 10^18^
VB effective DOS (cm^−3^)	1.8 × 10^19^	1.8 × 10^18^	1.8 × 10^19^
Electron mobility (cm^2^/V.s)	50	22	0.045
Hole mobility (cm^2^/V.s)	50	22	0.045
Donor density N_D_ (cm^−3^)	1 × 10^18^ *	7 × 10^15^	0
Acceptor density N_A_ (cm^−3^)	0	7 × 10^15^	1.8 × 10^18^
Defect type	SA	Neutral	Neutral
Energetic distribution	Single	Gaussian	Gaussian
Defect density (cm^−3^)	1 × 10^14^	1 × 10^14^	1 × 10^15^

* Variable field.

**Table 2:** A list of applied material parameters for interface defects incorporated in the simulations.

Parameters	HTL/FASnI_3_ Interface	FASnI_3_/ETL Interface
Defect type	Neutral	Single Acceptor (SA)
Capture cross s * variable field Section of electrons (cm^2^)	1 × 10^−18^	1 × 10^−18^
Capture cross-section of holes (cm^2^)	1 × 10^−18^	1 × 10^−17^
Reference for the defect energy level E_t_	Above E_v_	Above E
Energy with respect to reference (eV)	0.6	0.6
Total density (cm^−2^)	1 × 10^14^	1 × 10^14^ *

* Variable field.

**Table 3:** Comparison of key performance parameters for FASnI_3_ PSCs with and without the HTL.

PV Performance Parameters	(With HTL)	(Without HTL)
	Ag/PEDOT:PSS/FASnI_3_/C60/FTO	Ag/FASnI_3_/C60/FTO
PCE (%)	12.53	14.46
J_SC_ (mA/cm^2^)	20.57	22.50
V_OC_ (V)	0.77	0.78
FF (%)	78.25	82.09

**Table 4:** Comparison of the key photovoltaic parameters for the HTL-free FASnI_3_ structure before and after optimization.

PV Performance Parameters	(Before Optimization)	(After Optimization)
	Ag/FASnI_3_/C60/FTO	Ag/FASnI_3_/C60/FTO
PCE (%)	14.46	19.63
J_SC_ (mA/cm^2^)	22.50	27.86
V_OC_ (V)	0.78	0.87
FF (%)	82.09	81

## Data Availability

Data are available upon request.
